# Supplementary material for: Functional group based marine ecosystem assessment for the Bay of Biscay via elasticity analysis
Source: PeerJ. 2019 Aug 9;7:e7422. doi: 10.7717/peerj.7422 (PMC6690336; doi:10.7717/peerj.7422)
Supplement: Supplemental Information 1 — Functional group species membership, time series of catch and effort data, and supplementary results. [file peerj-07-7422-s001.pdf]

Supplementary Online Material: Functional Group Based Marine Ecosystem Assessment for the Bay of Biscay via Elasticity Analysis

Geoffrey R. Hosack[1] and Verena M. Trenkel[2]

[1]Commonwealth Scientific and Industrial Research Organisation, Hobart, Tasmania, Australia
[2]Ifremer, Nantes, France

Functional Group Membership and Data

Table S1: Functional group species membership.

Species	Functional Group
Ammodytes spp	Benthivore
Aspitrigla cuculus	Benthivore
Balistidae	Benthivore
Belone belone	Benthivore
Boops boops	Benthivore
Bothidae	Benthivore
Brachyura	Benthivore
Buglossidium luteum	Benthivore
Cancer pagurus	Benthivore
Caproidae	Benthivore
Carcinus maenas	Benthivore
Cephalopoda	Benthivore
Cepola macrophthalma	Benthivore
Chelidonichthys lastoviza	Benthivore
Chelidonichthys lucerna	Benthivore
Citharus linguatula	Benthivore
Coryphaenoides rupestris	Benthivore
Crangon crangon	Benthivore
Crustacea	Benthivore
Dicologlossa cuneata	Benthivore
Diplodus cervinus	Benthivore

Continued on next page

Table S1 – continued from previous page

Species	Functional Group
Diplodus sargus	Benthivore
Eutrigla gurnardus	Benthivore
Gaidropsarus spp	Benthivore
Galatheidae	Benthivore
Glyptocephalus cynoglossus	Benthivore
Haemulidae(=Pomadasyidae)	Benthivore
Helicolenus dactylopterus	Benthivore
Hippoglossoides platessoides	Benthivore
Homarus gammarus	Benthivore
Labridae	Benthivore
Labrus bergylta	Benthivore
Limanda limanda	Benthivore
Lithognathus mormyrus	Benthivore
Loliginidae	Benthivore
Maja squinado	Benthivore
Melanogrammus aeglefinus	Benthivore
Microchirus variegatus	Benthivore
Microstomus kitt	Benthivore
Mora moro	Benthivore
Moridae	Benthivore
Mugilidae	Benthivore
Mullus barbatus	Benthivore
Mullus spp	Benthivore
Necora puber	Benthivore
Nephrops norvegicus	Benthivore
Oblada melanura	Benthivore
Octopodidae	Benthivore
Octopus vulgaris	Benthivore
Pagellus acarne	Benthivore
Pagellus erythrinus	Benthivore
Palaemon longirostris	Benthivore
Palaemon serratus	Benthivore
Palinurus elephas	Benthivore
Palinurus mauritanicus	Benthivore
Parapenaeus longirostris	Benthivore
Phycis blennoides	Benthivore
Phycis phycis	Benthivore
Plectorhinchus mediterraneus	Benthivore
Pleuronectiformes	Benthivore
Sarpa salpa	Benthivore
Scyliorhinidae	Benthivore
Scyliorhinus canicula	Benthivore
Sepia elegans	Benthivore
Sepia officinalis	Benthivore
Sepia orbignyana	Benthivore
Sepiidae, Sepiolidae	Benthivore
Solea lascaris	Benthivore

Continued on next page

Table S1 – continued from previous page

**Species**	**Functional Group**
Solea senegalensis	Benthivore
Solea solea	Benthivore
Solea spp	Benthivore
Spondyliosoma cantharus	Benthivore
Trachinus draco	Benthivore
Trigla spp	Benthivore
Triglidae	Benthivore
Trisopterus luscus	Benthivore
Trisopterus minutus	Benthivore
Anarhichas lupus	Demersal Piscivore
Anguilla anguilla	Demersal Piscivore
Anguilla spp	Demersal Piscivore
Aphanopus carbo	Demersal Piscivore
Argyrosomus regius	Demersal Piscivore
Beryx spp	Demersal Piscivore
Brosme brosme	Demersal Piscivore
Centrophorus squamosus	Demersal Piscivore
Centroscymnus coelolepis	Demersal Piscivore
Centroscymnus crepidater	Demersal Piscivore
Conger conger	Demersal Piscivore
Dalatias licha	Demersal Piscivore
Dasyatis pastinaca	Demersal Piscivore
Deania calcea	Demersal Piscivore
Dentex dentex	Demersal Piscivore
Dentex spp	Demersal Piscivore
Dicentrarchus labrax	Demersal Piscivore
Dicentrarchus punctatus	Demersal Piscivore
Dicentrarchus spp	Demersal Piscivore
Epigonus telescopus	Demersal Piscivore
Epinephelus marginatus	Demersal Piscivore
Gadiformes	Demersal Piscivore
Gadus morhua	Demersal Piscivore
Galeorhinus galeus	Demersal Piscivore
Galeus melastomus	Demersal Piscivore
Hexanchus griseus	Demersal Piscivore
Hippoglossus hippoglossus	Demersal Piscivore
Hoplostethus atlanticus	Demersal Piscivore
Hydrolagus spp	Demersal Piscivore
Lepidopus caudatus	Demersal Piscivore
Lepidorhombus spp	Demersal Piscivore
Lepidorhombus whiffiagonis	Demersal Piscivore
Lophiidae	Demersal Piscivore
Lophius piscatorius	Demersal Piscivore
Lophius spp	Demersal Piscivore
Macrourus berglax	Demersal Piscivore
Merlangius merlangus	Demersal Piscivore
Merluccius merluccius	Demersal Piscivore

Continued on next page

Table S1 – continued from previous page

Species	Functional Group
Molva dypterygia	Demersal Piscivore
Molva macrophthalma	Demersal Piscivore
Molva molva	Demersal Piscivore
Molva spp	Demersal Piscivore
Mustelus spp	Demersal Piscivore
Myliobatis aquila	Demersal Piscivore
Pagellus bogaraveo	Demersal Piscivore
Pagrus pagrus	Demersal Piscivore
Platichthys flesus	Demersal Piscivore
Pleuronectes platessa	Demersal Piscivore
Pollachius pollachius	Demersal Piscivore
Pollachius virens	Demersal Piscivore
Polyprion americanus	Demersal Piscivore
Psetta maxima	Demersal Piscivore
Raja batis	Demersal Piscivore
Raja brachyura	Demersal Piscivore
Raja circularis	Demersal Piscivore
Raja clavata	Demersal Piscivore
Raja fullonica	Demersal Piscivore
Raja microocellata	Demersal Piscivore
Raja montagui	Demersal Piscivore
Raja naevus	Demersal Piscivore
Raja oxyrinchus	Demersal Piscivore
Raja spp	Demersal Piscivore
Raja undulata	Demersal Piscivore
Rajidae	Demersal Piscivore
Rajiformes	Demersal Piscivore
Reinhardtius hippoglossoides	Demersal Piscivore
Scophthalmus rhombus	Demersal Piscivore
Scorpaena scrofa	Demersal Piscivore
Scorpaena spp	Demersal Piscivore
Scorpaenidae	Demersal Piscivore
Scyliorhinus spp	Demersal Piscivore
Scyliorhinus stellaris	Demersal Piscivore
Scymnodon ringens	Demersal Piscivore
Sebastes spp	Demersal Piscivore
Sparidae	Demersal Piscivore
Sparus aurata	Demersal Piscivore
Squalidae	Demersal Piscivore
Squalidae, Scyliorhinidae	Demersal Piscivore
Squalus acanthias	Demersal Piscivore
Squalus spp	Demersal Piscivore
Squatina squatina	Demersal Piscivore
Torpedo marmorata	Demersal Piscivore
Torpedo spp	Demersal Piscivore
Trachyscorpia cristulata	Demersal Piscivore
Zeus faber	Demersal Piscivore

Continued on next page

Table S1 – continued from previous page

Species	Functional Group
Alopias spp	Pelagic Piscivore
Alopias vulpinus	Pelagic Piscivore
Auxis thazard	Pelagic Piscivore
Brama brama	Pelagic Piscivore
Cetorhinus maximus	Pelagic Piscivore
Euthynnus alletteratus	Pelagic Piscivore
Isurus oxyrinchus	Pelagic Piscivore
Katsuwonus pelamis	Pelagic Piscivore
Lamna nasus	Pelagic Piscivore
Lichia amia	Pelagic Piscivore
Loliginidae, Ommastrephidae	Pelagic Piscivore
Loligo spp	Pelagic Piscivore
Makaira nigricans	Pelagic Piscivore
Petromyzon marinus	Pelagic Piscivore
Prionace glauca	Pelagic Piscivore
Salmo salar	Pelagic Piscivore
Sarda sarda	Pelagic Piscivore
Selachimorpha (Pleurotremata)	Pelagic Piscivore
Tetrapturus albidus	Pelagic Piscivore
Thunnini	Pelagic Piscivore
Thunnus alalunga	Pelagic Piscivore
Thunnus albacares	Pelagic Piscivore
Thunnus atlanticus	Pelagic Piscivore
Thunnus obesus	Pelagic Piscivore
Thunnus thynnus	Pelagic Piscivore
Xiphias gladius	Pelagic Piscivore
Alosa alosa	Planktivore
Alosa fallax	Planktivore
Alosa spp	Planktivore
Argentina silus	Planktivore
Argentina sphyraena	Planktivore
Argentina spp	Planktivore
Clupea harengus	Planktivore
Engraulis encrasicolus	Planktivore
Illex coindetii	Planktivore
Illex illecebrosus	Planktivore
Illex spp	Planktivore
Mallotus villosus	Planktivore
Micromesistius poutassou	Planktivore
Osmerus eperlanus	Planktivore
Perciformes	Planktivore
Sardina pilchardus	Planktivore
Sardinella spp	Planktivore
Scomber scombrus	Planktivore
Scomber spp	Planktivore
Scomberesox saurus	Planktivore
Scombridae	Planktivore

Continued on next page

Table S1 – continued from previous page

Species	Functional Group
Scombroidei	Planktivore
Sprattus sprattus	Planktivore
Trachurus mediterraneus	Planktivore
Trachurus spp	Planktivore
Trachurus trachurus	Planktivore
Trisopterus esmarkii	Planktivore

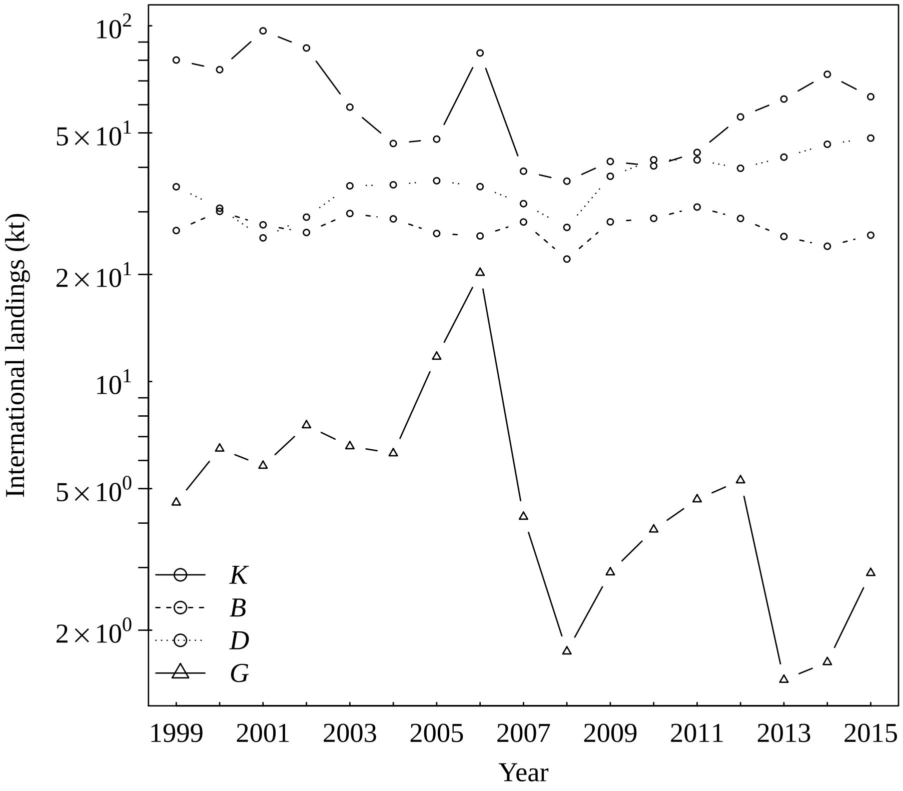

Figure S1: The international landings for ICES division 8a and 8b by functional group.

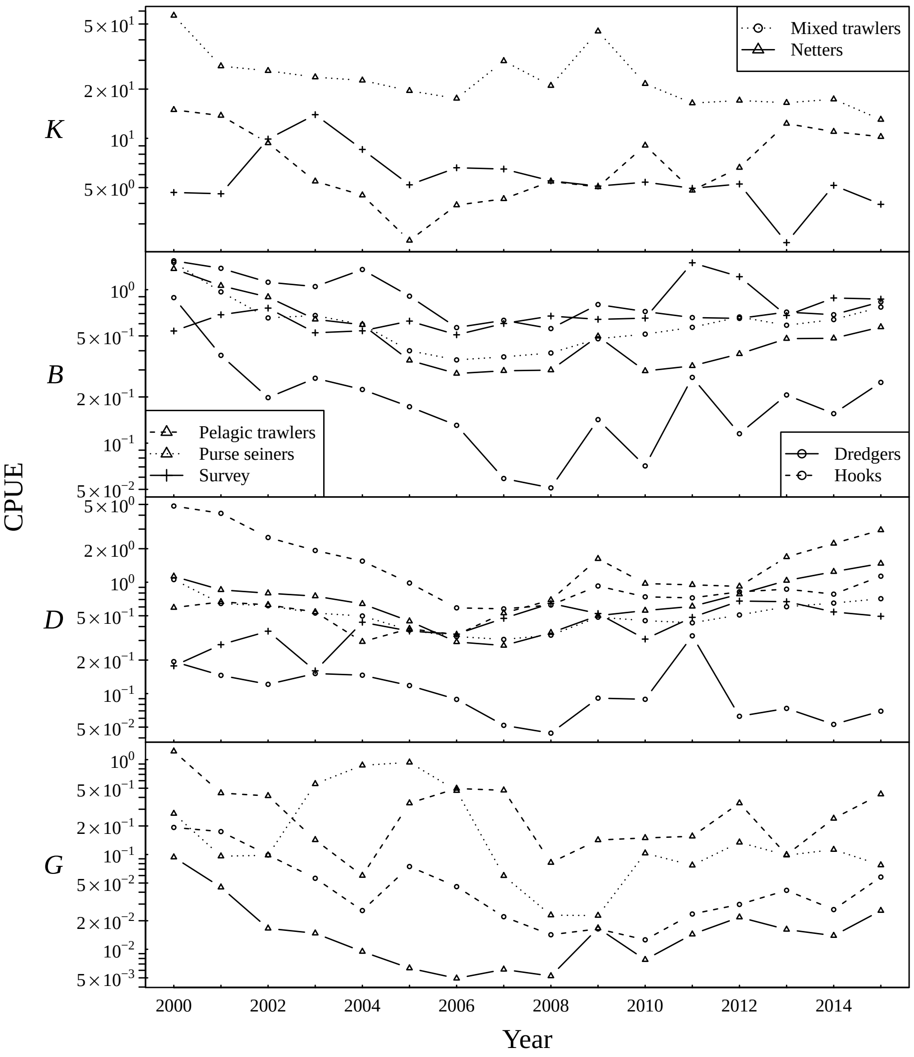

Figure S2: Time series of catch per unit effort (metric tonnes per day of fishing) for each of the French fisheries by functional group (see legend above) and the survey mean (metric tonnes per km^2).

Model Results

Table S2: Posterior quantiles of estimated log catchability ($\log q_{ik}$) for K: planktivores, B: benthivores, D: demersal piscivores, and G: pelagic piscivores.

Gear	FG	2.5%	50%	97.5%
Dredgers	B	−6.17	−5.84	−5.53
Dredgers	D	−7.11	−6.75	−6.42
Hooks	D	−4.64	−4.29	−3.98
Hooks	G	−6.47	−5.95	−5.52
Mixed trawlers	B	−4.84	−4.62	−4.43
Mixed trawlers	D	−5.40	−5.11	−4.90
Netters	B	−5.11	−4.81	−4.53
Netters	D	−5.20	−4.86	−4.56
Netters	G	−7.51	−7.01	−6.58
Pelagic trawlers	K	−3.40	−2.92	−2.49
Pelagic trawlers	D	−5.05	−4.66	−4.30
Pelagic trawlers	G	−4.70	−4.16	−3.69
Potters	B	−4.52	−4.26	−4.04
Purse seiners	K	−2.25	−1.73	−1.23
Purse seiners	G	−5.33	−4.73	−4.22
Survey	K	−3.57	−3.11	−2.69
Survey	B	−4.78	−4.43	−4.11
Survey	D	−5.73	−5.35	−5.00

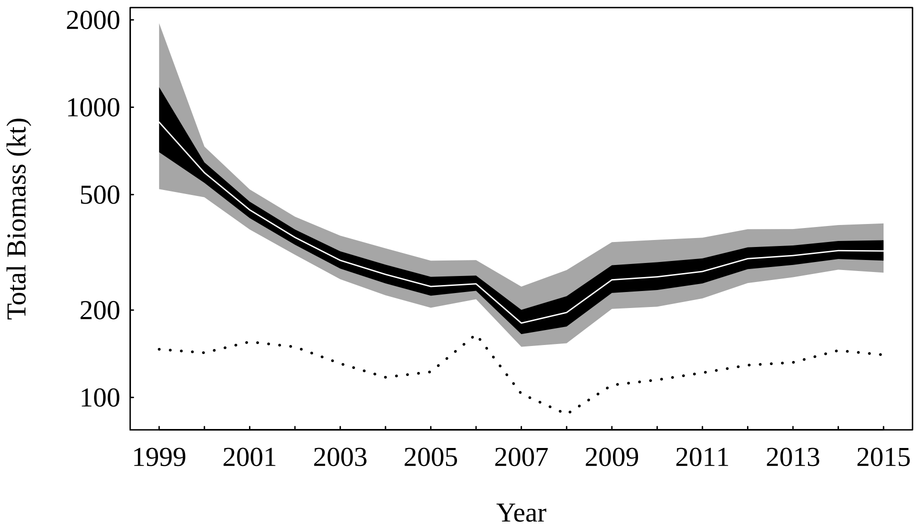

Figure S3: Latent state estimates for total biomass. The dotted line is the total annual harvest for each functional group. Posterior medians given by white lines, 50% central credible intervals by black polygons and 90% central CIs by grey polygons. Note logarithmic y-axes.

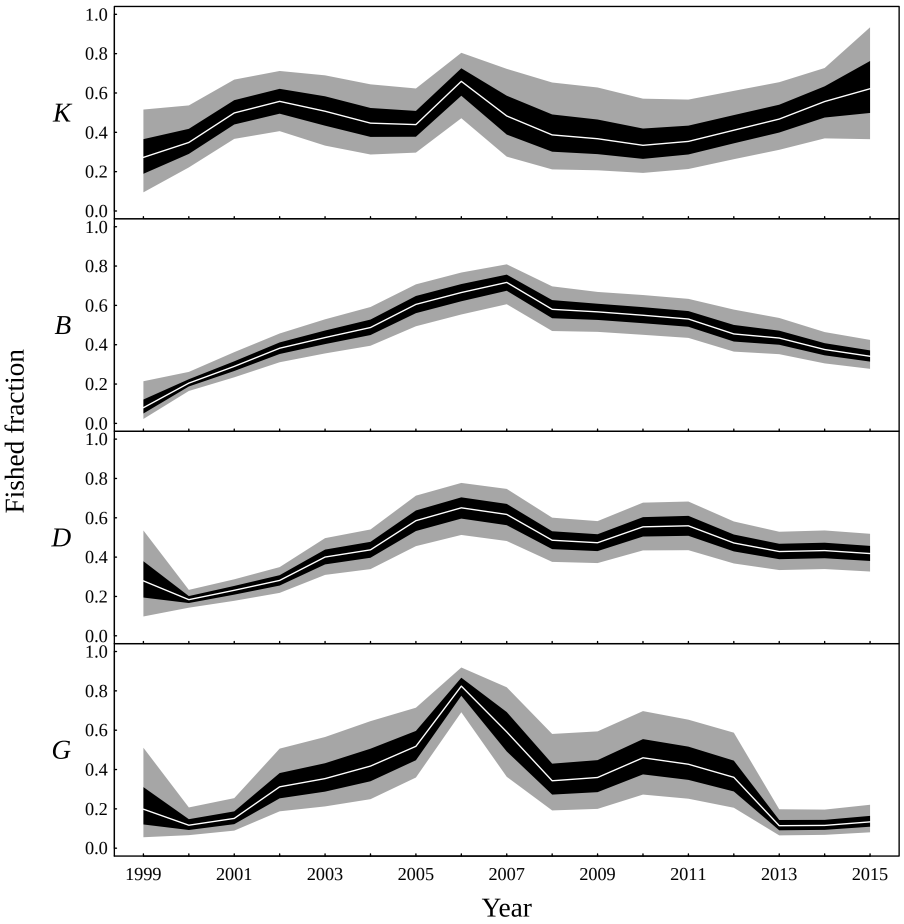

Figure S4: Estimated annual fished fraction ($F_i(t)$) of fishable biomass for K: planktivores, B: benthivores, D: demersal piscivores, and G: pelagic piscivores. Posterior medians given by white lines, 50% central credible intervals by black polygons and 90% central CIs by grey polygons.

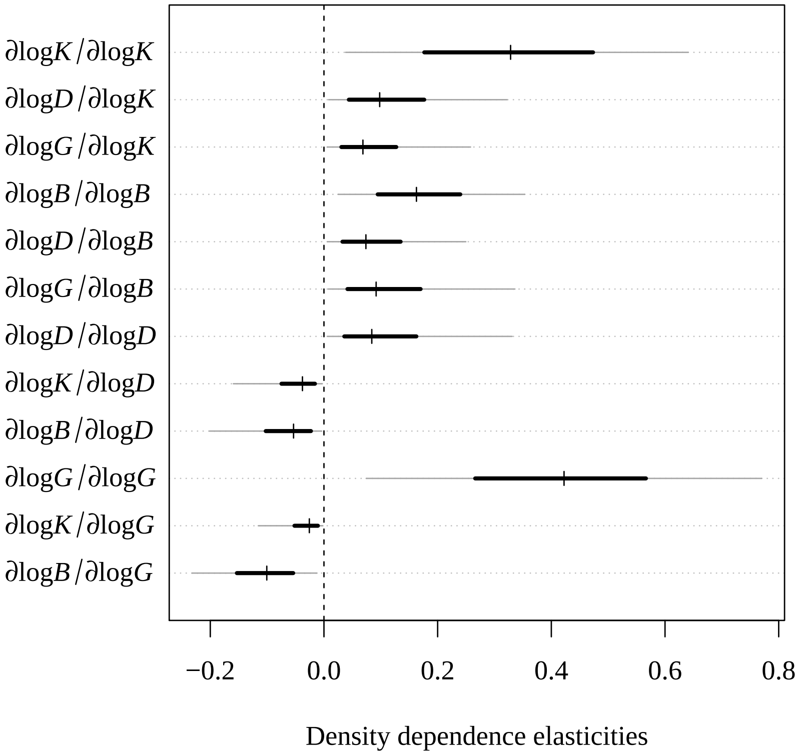

Figure S5: The elasticities of the one year look-ahead predictions to changes in the previous year's biomass for K: planktivores, B: benthivores, D: demersal piscivores, and G: pelagic piscivores. Posterior medians given by hashes, 50% central CIs by black segments and 90% central CIs by grey segments.

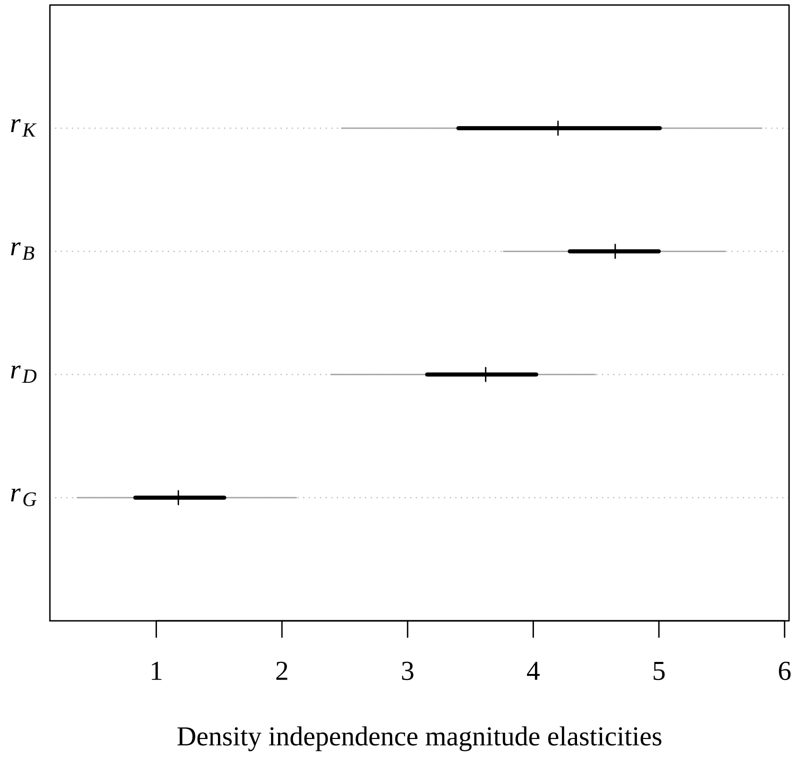

Figure S6: Elasticities of the one year look-ahead predictions of each functional group to changes in the magnitude of its density independent growth parameter, $|r_i|$, for K: planktivores, B: benthivores, D: demersal piscivores, and G: pelagic piscivores. Posterior medians given by hashes, 50% central CIs by black segments and 90% central CIs by grey segments.

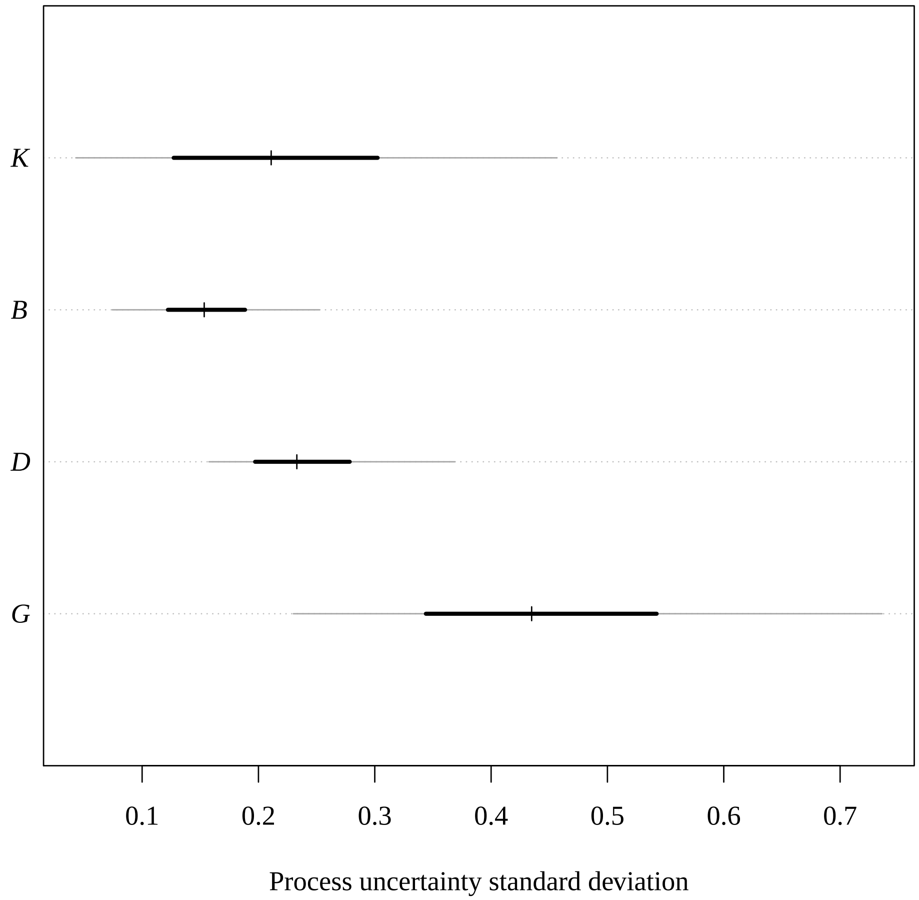

Figure S7: Magnitude of process uncertainty (standard deviation of process error) for K: planktivores, B: benthivores, D: demersal piscivores, and G: pelagic piscivores. Posterior medians given by hashes, 50% central CIs by black segments and 90% central CIs by grey segments.

Table S3: Posterior interquartile distance for the elasticities of the long term stationary distribution means or quantiles with respect to the unfished fractions. The table entries correspond to the entries of the elasticity matrix $-A^{-1}$.

	K	B	D	G
K	0.67	0.02	0.10	0.12
B	0.05	0.20	0.11	0.24
D	0.23	0.13	0.15	0.04
G	0.28	0.26	0.03	0.91

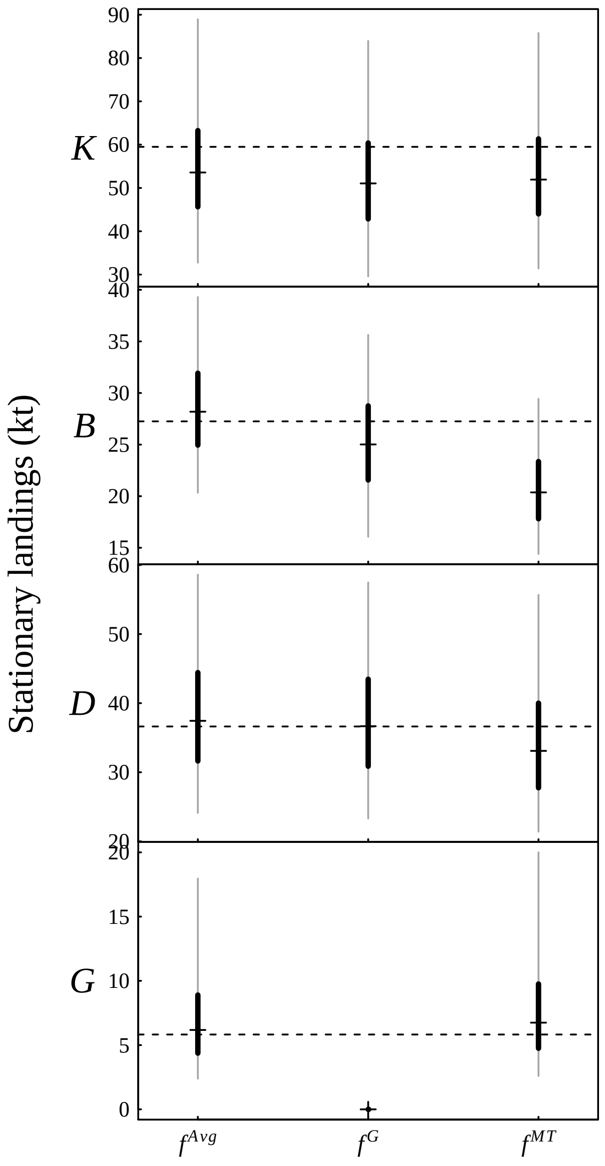

Figure S8: Plotted are the posterior stationary distribution of annual landings under the three management scenarios with non-zero fishing mortalities for K: planktivores, B: benthivores, D: demersal piscivores, and G: pelagic piscivores. Posterior medians given by hashes, 50% central CIs by black segments and 90% central CIs by grey segments. The average landings for years 2000-2015 are shown by horizontal dashed lines. No landings occur for the scenario with no fishing (f_0).
