# Supplementary material for: Functional group based marine ecosystem assessment for the Bay of Biscay via elasticity analysis
Source: PeerJ. 2019 Aug 9;7:e7422. doi: 10.7717/peerj.7422 (PMC6690336; doi:10.7717/peerj.7422)
Supplement: Supplemental Information 2 — This vignette demonstrates use of the rBiscayCodeData R package. [file peerj-07-7422-s002.pdf]

rBiscayCodeData: Supplementary Code and Data for Estimation of Multivariate Bayesian State Space Model by Markov Chain Monte Carlo

Geoffrey R. Hosack
Commonwealth Scientific and Industrial Research Organisation
Hobart, Tasmania, Australia

Verena M. Trenkel
Ifremer
Nantes, France

Abstract

The R package **rBiscayCodeData** provides the code and data that supports the Bayesian state space model of (Hosack and Trenkel 2019). The species membership by functional group, original sources and filtering steps are as described in (Hosack and Trenkel 2019), as are the model formulation and analysis. The estimation uses Markov chain Monte Carlo as implemented by JAGS, which can be obtained at `http://mcmc-jags.sourceforge.net`. The analysis depends on the R port of JAGS through package **rjags** (Plummer 2016).

Keywords: R.

This vignette describes the code and data for Hosack and Trenkel (2019). See Hosack and Trenkel (2019) for a description of the statistical model. The results in this paper were obtained using R 3.5.1 with the **rBiscayCodeData** 1.0 package. R itself and all packages used are available from the Comprehensive R Archive Network (CRAN) at `https://CRAN.R-project.org/`. It is assumed that the user is able to install R and the **rBiscayCodeData** package. The necessary R documentation is available on the R homepage at `https://www.r-project.org/`.

The following code chunk loads the package and also the catch and effort data aggregated to the level of the functional groups. The data are as described in Hosack and Trenkel (2019). The original data sources were as follows: the survey biomass data were sourced from Système d'Information Halieutique, the French commerical catch and effort data from the Direction des pêches maritimes et de l'aquaculture, and the international landings data from the International Council for the Exploration of the Seas (`http://www.ices.dk/marine-data/dataset-collections`). See `?data_for_analysis` for further details.

```
R> library(rBiscayCodeData)
R> data("data_for_analysis")
```

Create list of data for input to JAGS.

```
R> jags.data <- list(log.landings = log(landings),
+                    log.cpue.K = log(cpue.K), log.cpue.B = log(cpue.B),
+                    log.cpue.D = log(cpue.D), log.cpue.G = log(cpue.G),
+                    TT = TT, n.g = n.g, landings.1998 = landings.1998,
+                    mean.xstar = mean.landings.1950.1998
+ )
```

Set random number generator seed and define a function to generate initial values for the MCMC runs.

```
R> set.seed(1001)
R> jags.inits <- function() {
+
+   # initial values for latent states:
+   # draw from a Pareto distribution with minimum value given by landings
+   # and index parameter of 2
+   # then transform to log scale
+   log.x <- log(landings*exp(rexp(nrow(landings)*ncol(landings), rate = 2)))
+
+   a.KK <- runif(1, 0, 1)
+   a.BB <- runif(1, 0, 1)
+   a.DD <- runif(1, 0, 1)
+   a.GG <- runif(1, 0, 1)
+
+   list(
+
+     # intra-functional group density dependence
+     a.KK = a.KK,
+     a.BB = a.BB,
+     a.DD = a.DD,
+     a.GG = a.GG,
+
+     # density independence
+     r.K = rnorm(1, a.KK*log(mean.landings.1950.1998["K"]), a.KK),
+     r.B = rnorm(1, a.BB*log(mean.landings.1950.1998["B"]), a.BB),
+     r.D = rnorm(1, a.DD*log(mean.landings.1950.1998["D"]), a.DD),
+     r.G = rnorm(1, a.GG*log(mean.landings.1950.1998["G"]), a.GG),
+
+     # inter-functional group density dependence
+     a.BD = runif(1, 0, 1),
+     a.KD = runif(1, 0, 1),
+     a.BG = runif(1, 0, 1),
+     a.KG = runif(1, 0, 1),
+
+     a.DB = runif(1, 0, 1),
+     a.DK = runif(1, 0, 1),
+     a.GB = runif(1, 0, 1),
```

```
+      a.GK = runif(1, 0, 1),
+
+      # process noise
+      prec.eta = rlnorm(4, -1.760326, 1.061352)^-2,
+
+      # observation noise - French gears and survey
+      prec.sigma.DD = rlnorm(1, -1.760326, 1.061352)^(-2), # dredge
+      prec.sigma.HH = rlnorm(1, -1.760326, 1.061352)^(-2), # hook
+      prec.sigma.MT = rlnorm(1, -1.760326, 1.061352)^(-2), # mixed trawl
+      prec.sigma.NN = rlnorm(1, -1.760326, 1.061352)^(-2), # net
+      prec.sigma.PT = rlnorm(1, -1.760326, 1.061352)^(-2), # pelagic trawl
+      prec.sigma.PP = rlnorm(1, -1.760326, 1.061352)^(-2), # pot
+      prec.sigma.PS = rlnorm(1, -1.760326, 1.061352)^(-2), # purse seine
+      prec.sigma.SS = rlnorm(1, -1.760326, 1.061352)^(-2), # survey
+
+      # French gear catchabilities
+      log.q.K = colMeans(log(cpue.K)),
+      log.q.B = colMeans(log(cpue.B)),
+      log.q.D = colMeans(log(cpue.D)),
+      log.q.G = colMeans(log(cpue.G)),
+
+      # log latent states
+      log.x = log.x,
+
+      # z = -log(F(t)), F(t) ~ U(0, 1)
+      z = -log(matrix(runif(TT*4), nrow = TT, ncol = 4))
+
+    )
+  }
```

Store initial values in a list of lists object, including seeds for the JAGS random number generator.

```
R> # chain initialisations
R> n.chains <- 5
R> inits.ls <- list(
+    jags.inits(),
+    jags.inits(),
+    jags.inits(),
+    jags.inits(),
+    jags.inits()
+  )
R> # RNG initialisation for jags
R> for (i in 1:n.chains) {
+    inits.ls[[i]]$.RNG.name <- "base::Mersenne-Twister"
+    inits.ls[[i]]$.RNG.seed <- i*1000
+  }
```

The model is defined for JAGS and stored as a character vector.

```
R> ssm.jag <-
+  "
+  model
+  {
+
+    # t = 0 is 1999 (tt = 1); t = 1 is 2000 (tt = 2); t = 16 is 2015 (TT = 17)
+    # time applies to log.x, which extends from 1999--2015
+    # time index for landings and cpue matrices are adjusted as needed
+
+    # French landings and survey observation models -- log cpue,
+    # t = 1, ..., T --> 2000:2015
+    # gears: Dredgers (DD), Hooks (HH), Mixed trawlers (MT), Netters (NN),
+    # Pelagic trawlers (PT), Potters (PP), Purse seiners (PS), Survey (SS)
+    # FGs: K, B, D, G
+    for (tt in 2:TT) {
+
+      # by FG within gear type
+      # cpue matrices do not include t = 0 (tt = 1) and
+      # have one less row than log.x
+      # so for cpue subtract one from tt for same year as log.x
+
+      # 1) dredgers
+      log.cpue.B[tt - 1, 1] ~ dnorm(log.q.B[1] + log.x[tt, 2], prec.sigma.DD)
+      log.cpue.D[tt - 1, 1] ~ dnorm(log.q.D[1] + log.x[tt, 3], prec.sigma.DD)
+
+      # 2) hooks
+      log.cpue.D[tt - 1, 2] ~ dnorm(log.q.D[2] + log.x[tt, 3], prec.sigma.HH)
+      log.cpue.G[tt - 1, 1] ~ dnorm(log.q.G[1] + log.x[tt, 4], prec.sigma.HH)
+
+      # 3) mixed trawlers
+      log.cpue.B[tt - 1, 2] ~ dnorm(log.q.B[2] + log.x[tt, 2], prec.sigma.MT)
+      log.cpue.D[tt - 1, 3] ~ dnorm(log.q.D[3] + log.x[tt, 3], prec.sigma.MT)
+
+      # 4) netters
+      log.cpue.B[tt - 1, 3] ~ dnorm(log.q.B[3] + log.x[tt, 2], prec.sigma.NN)
+      log.cpue.D[tt - 1, 4] ~ dnorm(log.q.D[4] + log.x[tt, 3], prec.sigma.NN)
+      log.cpue.G[tt - 1, 2] ~ dnorm(log.q.G[2] + log.x[tt, 4], prec.sigma.NN)
+
+      # 5) pelagic trawlers
+      log.cpue.K[tt - 1, 1] ~ dnorm(log.q.K[1] + log.x[tt, 1], prec.sigma.PT)
+      log.cpue.D[tt - 1, 5] ~ dnorm(log.q.D[5] + log.x[tt, 3], prec.sigma.PT)
+      log.cpue.G[tt - 1, 3] ~ dnorm(log.q.G[3] + log.x[tt, 4], prec.sigma.PT)
+
+      # 6) potters
+      log.cpue.B[tt - 1, 4] ~ dnorm(log.q.B[4] + log.x[tt, 2], prec.sigma.PP)
```

```
+
+       # 7) purse seiners
+       log.cpue.K[tt - 1, 2] ~ dnorm(log.q.K[2] + log.x[tt, 1], prec.sigma.PS)
+       log.cpue.G[tt - 1, 4] ~ dnorm(log.q.G[4] + log.x[tt, 4], prec.sigma.PS)
+
+       # 8) survey
+       log.cpue.K[tt - 1, 3] ~ dnorm(log.q.K[3] + log.x[tt, 1], prec.sigma.SS)
+       log.cpue.B[tt - 1, 5] ~ dnorm(log.q.B[5] + log.x[tt, 2], prec.sigma.SS)
+       log.cpue.D[tt - 1, 6] ~ dnorm(log.q.D[6] + log.x[tt, 3], prec.sigma.SS)
+
+     }
+
+     # latent states t = 0
+     for (i in 1:4) {
+       log.x[1, i] ~ dnorm(log(landings.1998[i]), 0.308642)
+     }
+
+     # latent states t = 1, ..., T
+     for (tt in 2:TT) {
+
+       # location parameters: depends on log biomass from previous year
+       # z = -log(F)
+       mu[tt - 1, 1] <- log.x[tt - 1, 1] + r.K + log(1 - exp(-z[tt - 1, 1])) +
+         - a.KK*log.x[tt - 1, 1] - a.KG*log.x[tt - 1, 4] - a.KD*log.x[tt - 1, 3]
+       mu[tt - 1, 2] <- log.x[tt - 1, 2] + r.B + log(1 - exp(-z[tt - 1, 2])) +
+         - a.BB*log.x[tt - 1, 2] - a.BG*log.x[tt - 1, 4] - a.BD*log.x[tt - 1, 3]
+       mu[tt - 1, 3] <- log.x[tt - 1, 3] + r.D + log(1 - exp(-z[tt - 1, 3])) +
+         - a.DD*log.x[tt - 1, 3] + a.DK*log.x[tt - 1, 1] + a.DB*log.x[tt - 1, 2]
+       mu[tt - 1, 4] <- log.x[tt - 1, 4] + r.G + log(1 - exp(-z[tt - 1, 4])) +
+         - a.GG*log.x[tt - 1, 4] + a.GK*log.x[tt - 1, 1] + a.GB*log.x[tt - 1, 2]
+
+       # transition and latent states
+       for (i in 1:4) {
+         log.x[tt, i] ~ dnorm(mu[tt - 1, i], prec.eta[i])
+       }
+
+     }
+
+     # fishing fraction and landings t = 0, ..., T
+     # log landings reported with error
+     # z = -log(F)
+     for (tt in 1:TT) {
+       log.landings[tt, 1] ~ dnorm(sum(-z[tt, 1], log.x[tt, 1]), 1000)
+       log.landings[tt, 2] ~ dnorm(sum(-z[tt, 2], log.x[tt, 2]), 1000)
+       log.landings[tt, 3] ~ dnorm(sum(-z[tt, 3], log.x[tt, 3]), 1000)
+       log.landings[tt, 4] ~ dnorm(sum(-z[tt, 4], log.x[tt, 4]), 1000)
+     }
```

```
+
+     # Given uniform prior for F, transformation z = -log(F)
+     # induces Exp(1) prior for z
+     for (tt in 1:TT) {
+       z[tt, 1] ~ dexp(1)
+       z[tt, 2] ~ dexp(1)
+       z[tt, 3] ~ dexp(1)
+       z[tt, 4] ~ dexp(1)
+     }
+
+     # process model parameters
+
+     # process error
+     # for parameterisation see observation error below
+     for (i in 1:4) {
+       prec.eta[i] ~ dlnorm(3.520652, 0.2219326)
+       eta[i] <- prec.eta[i]^-0.5
+     }
+
+     # density independent rate of growth
+     r.K ~ dnorm(a.KK*log(mean.xstar[1]), a.KK^-2)
+     r.B ~ dnorm(a.BB*log(mean.xstar[2]), a.BB^-2)
+     r.D ~ dnorm(a.DD*log(mean.xstar[3]), a.DD^-2)
+     r.G ~ dnorm(a.GG*log(mean.xstar[4]), a.GG^-2)
+
+     # intraspecific density dependence (magnitude)
+     a.KK ~ dunif(0, 1)
+     a.BB ~ dunif(0, 1)
+     a.DD ~ dunif(0, 1)
+     a.GG ~ dunif(0, 1)
+
+     # interspecific density dependence (magnitude)
+     a.BD ~ dunif(0, 1)
+     a.KD ~ dunif(0, 1)
+     a.BG ~ dunif(0, 1)
+     a.KG ~ dunif(0, 1)
+
+     a.DB ~ dunif(0, 1)
+     a.DK ~ dunif(0, 1)
+     a.GB ~ dunif(0, 1)
+     a.GK ~ dunif(0, 1)
+
+     # observation error
+     # dlnorm prior for sigma is mu =   -1.760326, sigma = 1.061352,
+     # prec = sigma^-2 = 0.8877306
+     # induced dlnorm prior for precision is:
+     # mu' = -2*mu = 3.520652 and prec' = (-2)^(-2)*prec = 0.2219326
```

```
+    prec.sigma.DD ~ dlnorm(3.520652, 0.2219326)
+    sigma.DD <- prec.sigma.DD^-0.5
+    prec.sigma.HH ~ dlnorm(3.520652, 0.2219326)
+    sigma.HH <- prec.sigma.HH^-0.5
+    prec.sigma.MT ~ dlnorm(3.520652, 0.2219326)
+    sigma.MT <- prec.sigma.MT^-0.5
+    prec.sigma.NN ~ dlnorm(3.520652, 0.2219326)
+    sigma.NN <- prec.sigma.NN^-0.5
+    prec.sigma.PT ~ dlnorm(3.520652, 0.2219326)
+    sigma.PT <- prec.sigma.PT^-0.5
+    prec.sigma.PP ~ dlnorm(3.520652, 0.2219326)
+    sigma.PP <- prec.sigma.PP^-0.5
+    prec.sigma.PS ~ dlnorm(3.520652, 0.2219326)
+    sigma.PS <- prec.sigma.PS^-0.5
+    prec.sigma.SS ~ dlnorm(3.520652, 0.2219326)
+    sigma.SS <- prec.sigma.SS^-0.5
+
+    # French catchabilities
+    # K
+    for (j in 1:n.g[1]) {
+      log.q.K[j] ~ dnorm(-log(mean.xstar[1]), 0.08549639)
+    }
+    # B
+    for (j in 1:n.g[2]) {
+      log.q.B[j] ~ dnorm(-log(mean.xstar[2]), 0.08549639)
+    }
+    # D
+    for (j in 1:n.g[3]) {
+      log.q.D[j] ~ dnorm(-log(mean.xstar[3]), 0.08549639)
+    }
+    # G
+    for (j in 1:n.g[4]) {
+      log.q.G[j] ~ dnorm(-log(mean.xstar[4]), 0.08549639)
+    }
+
+  }"
```

Load package **rjags** and initialise model. The following code chunk is unevaluated because of the time required for the simulation to run. The user time was 15 minutes using an Intel(R) Core(TM) i7–8650U CPU (1.90 GHz) processor.

```
R> library(rjags)
R> system.time(
+    mcmc.mod <- jags.model(
+      file = textConnection(ssm.jag),
+      data = jags.data,
+      inits = inits.ls,
```

```
+       n.chains = n.chains,
+       n.adapt = 500000
+     )
+   )
```

Specify variable names to be traced.

```
R> l <- inits.ls[[1]]
R> v.trace <- names(l)
R> v.trace <- v.trace[v.trace != ".RNG.name" & v.trace != ".RNG.seed"]
R> v.trace[grep("prec.eta", v.trace)] <- "eta"
R> v.trace <- gsub("prec.sigma", "sigma", v.trace)
```

The following code chunk is unevaluated because of the time required for the simulation to run. The user time was 16 minutes using an Intel(R) Core(TM) i7–8650U CPU (1.90 GHz) processor.

```
R> system.time(
+    mcmc.res <- coda.samples(
+       model = mcmc.mod,
+       variable.names = v.trace,
+       thin = 10,
+       n.iter = 500000
+     )
+   )
```

Acknowledgments

The authors thank the International Council for the Exploration of the Seas for the landings data, the Direction des pêches maritimes et de l'aquaculture for the French commercial catch and effort data, and Système d'Information Halieutique for the fishery independent scientific survey data.

References

Hosack GR, Trenkel VM (2019). "Functional Group-based Marine Ecosystem Assessment for the Bay of Biscay via Elasticity Analysis." *Submitted to PeerJ*.

Plummer M (2016). *rjags: Bayesian Graphical Models using MCMC*. R package version 4-6, URL https://CRAN.R-project.org/package=rjags.

Affiliation:

Geoffrey R. Hosack
Commonwealth Scientific and Industrial Research Organisation

Hobart, Tasmania, Australia 7001
E-mail: geoff.hosack@csiro.au
and
Verena M. Trenkel
Ifremer
Nantes, France
verena.trenkel@ifremer.fr
