# Supplementary figures and images for: Functional group based marine ecosystem assessment for the Bay of Biscay via elasticity analysis
Source: PeerJ. 2019 Aug 9;7:e7422. doi: 10.7717/peerj.7422 (PMC6690336; doi:10.7717/peerj.7422)

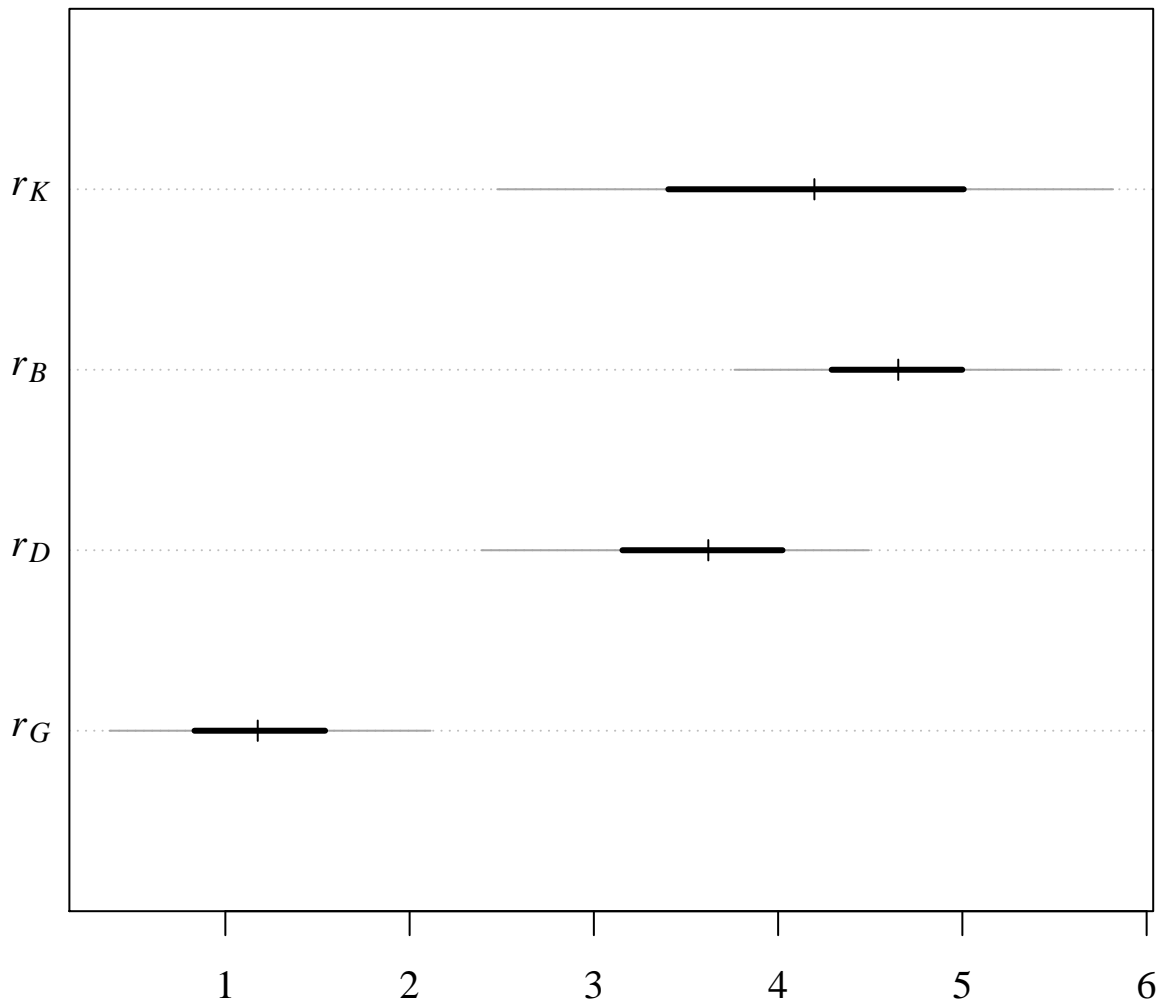

Density independence magnitude elasticities

Supplement: Supplemental Information 4 [file peerj-07-7422-s004.pdf]

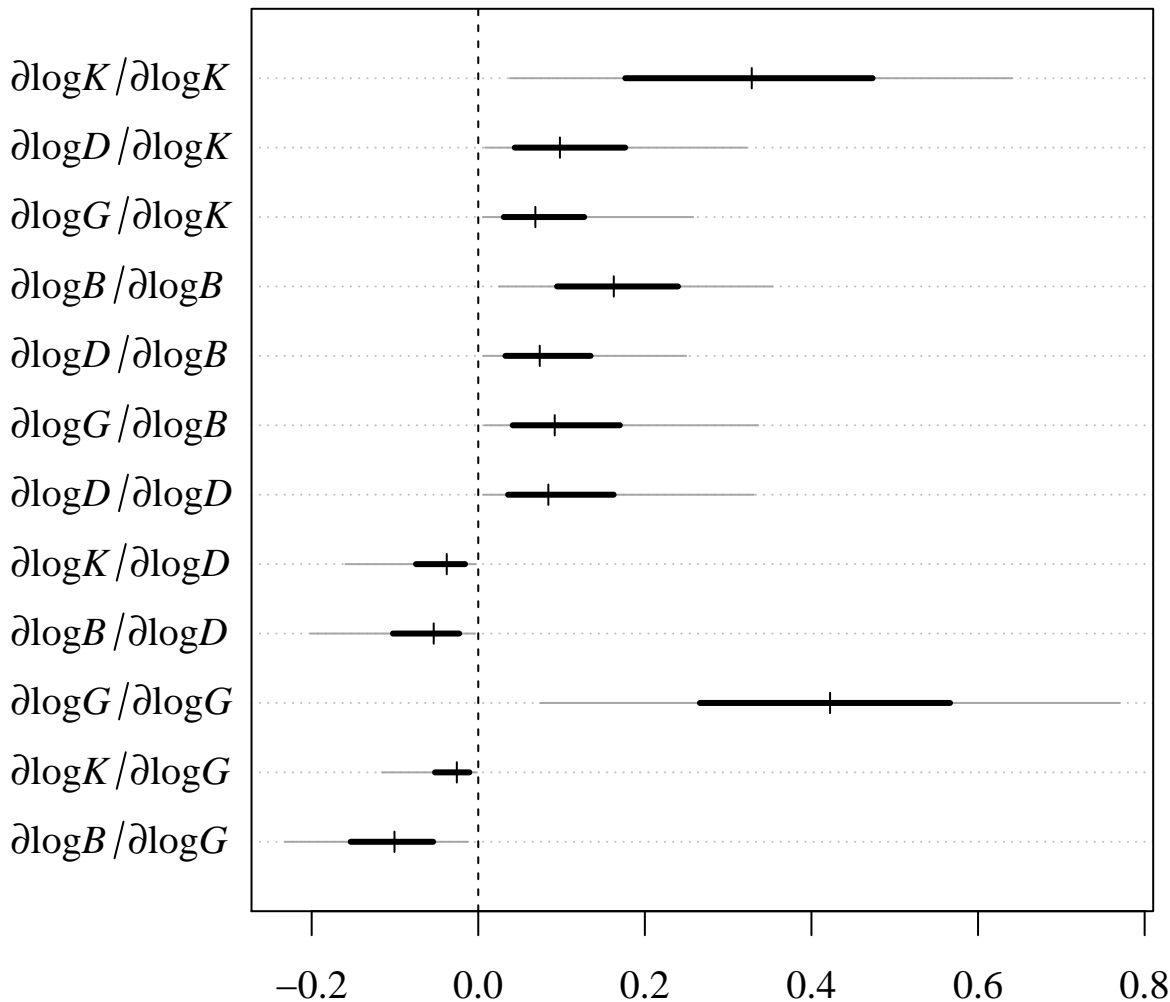

Density dependence elasticities

Supplement: Supplemental Information 5 [file peerj-07-7422-s005.pdf]

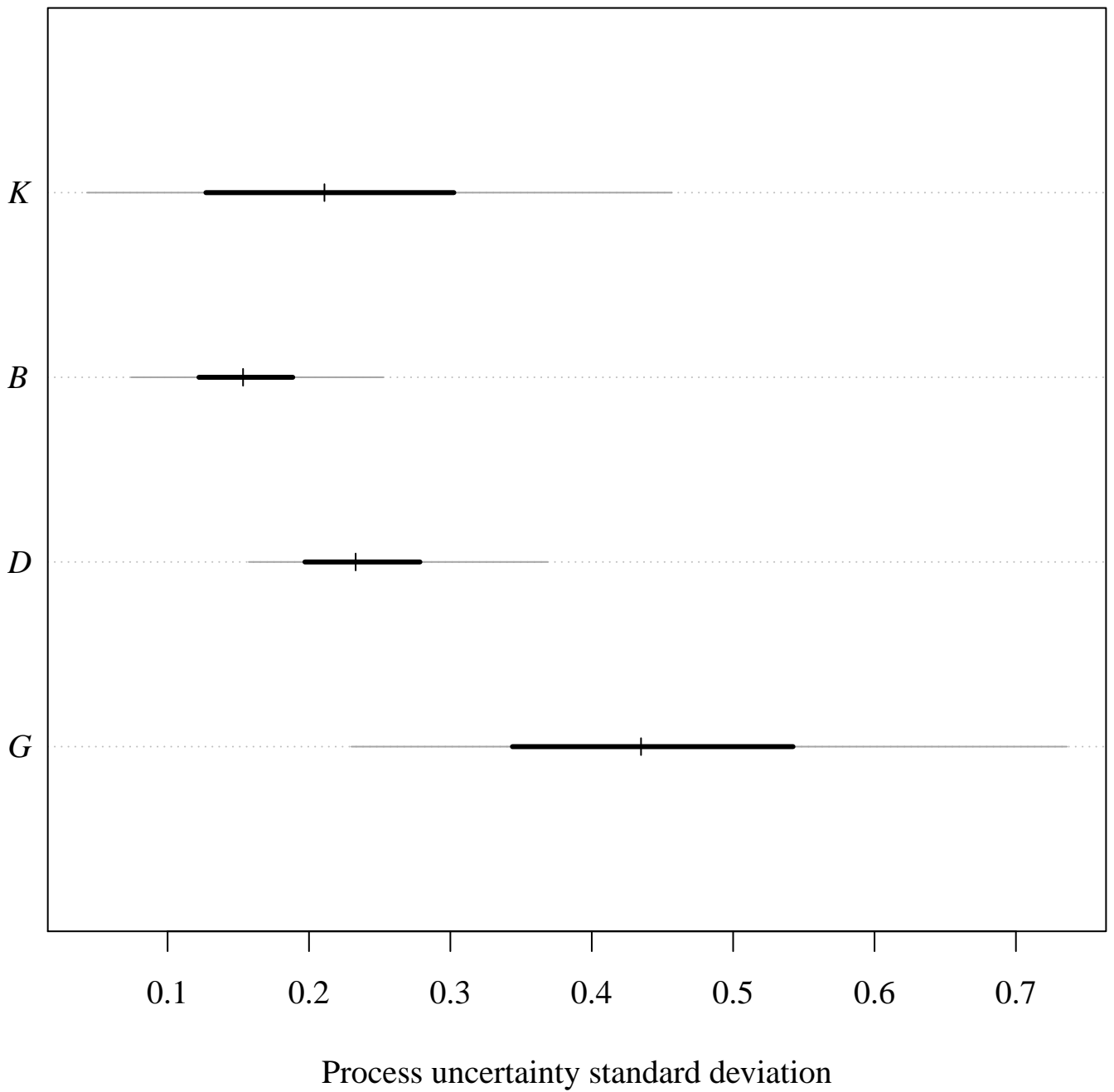

Supplement: Supplemental Information 6 [file peerj-07-7422-s006.pdf]

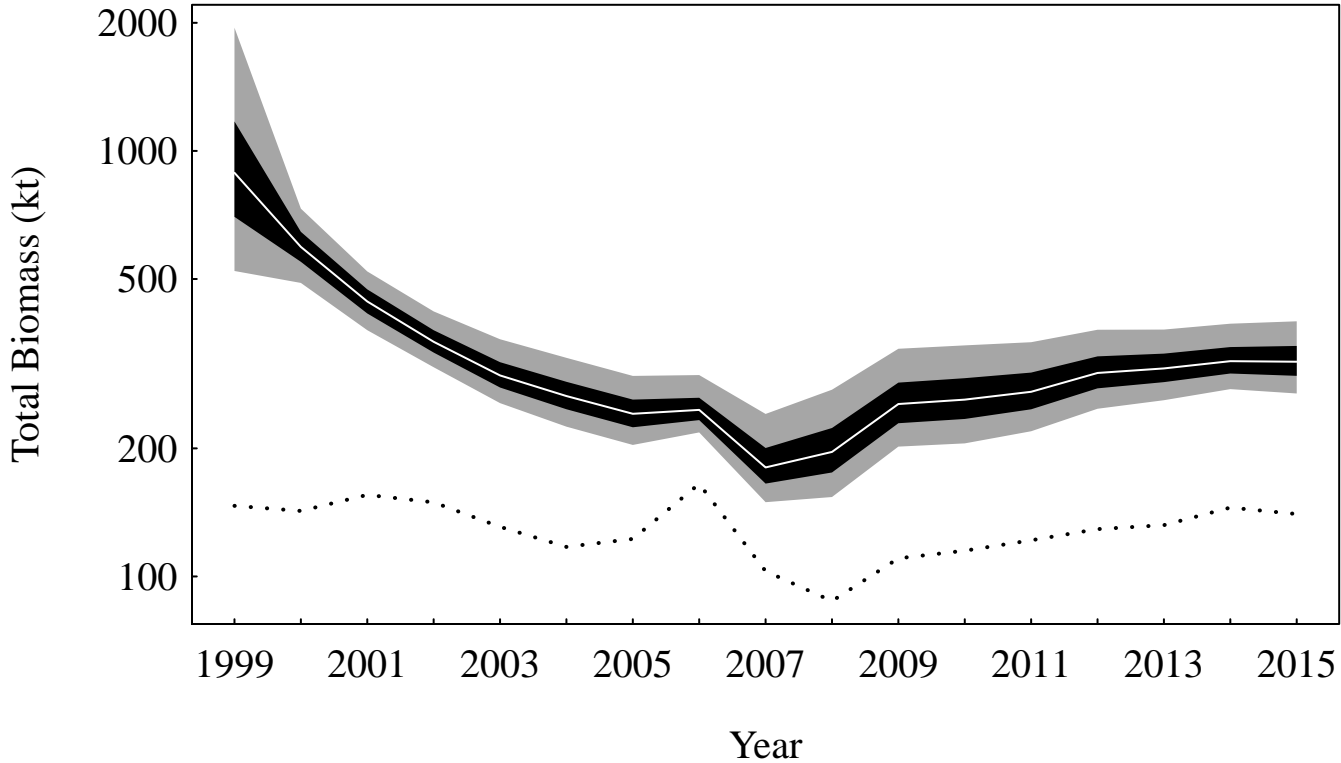

Supplement: Supplemental Information 7 [file peerj-07-7422-s007.pdf]

Fished fraction

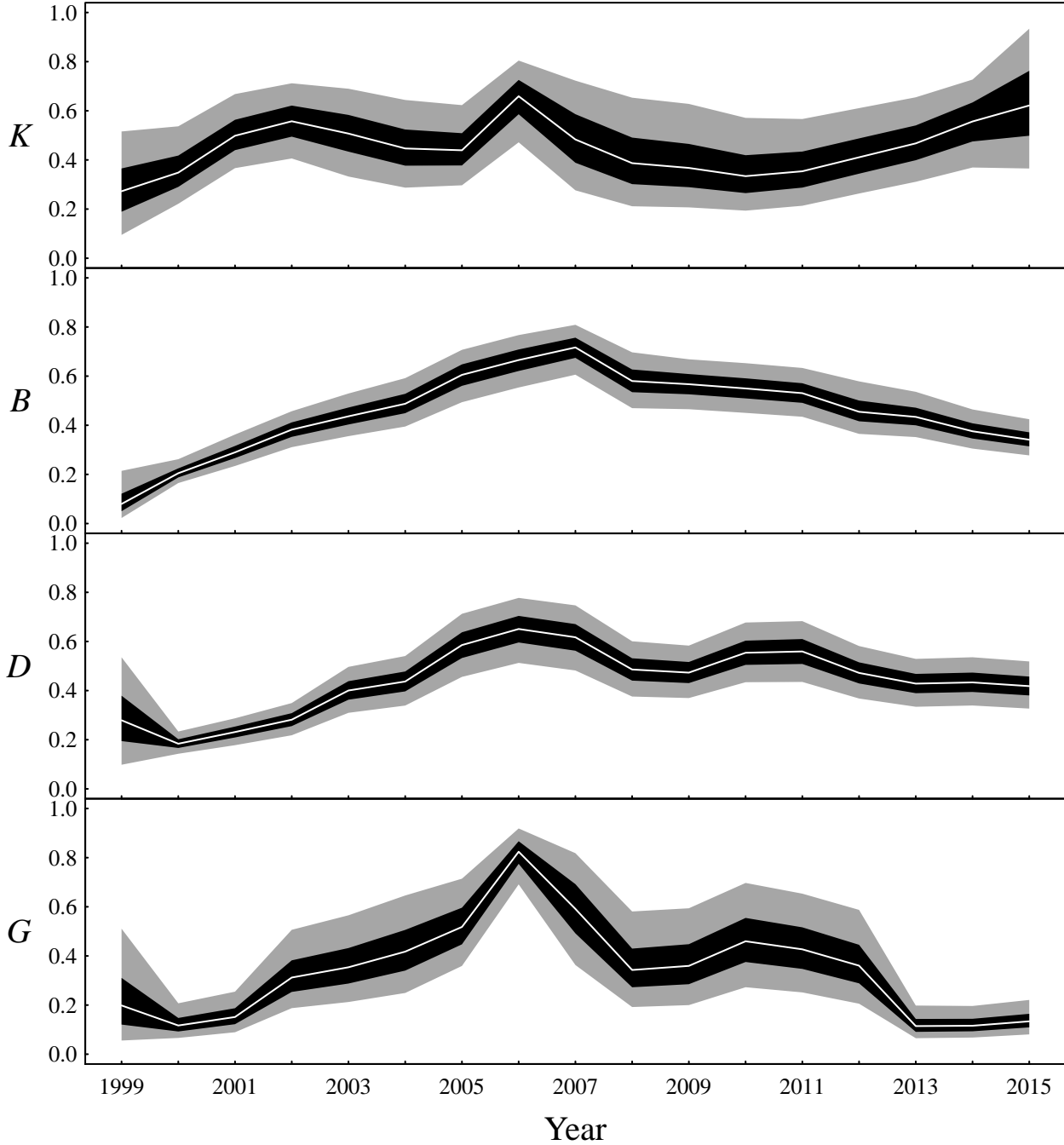

Supplement: Supplemental Information 8 [file peerj-07-7422-s008.pdf]

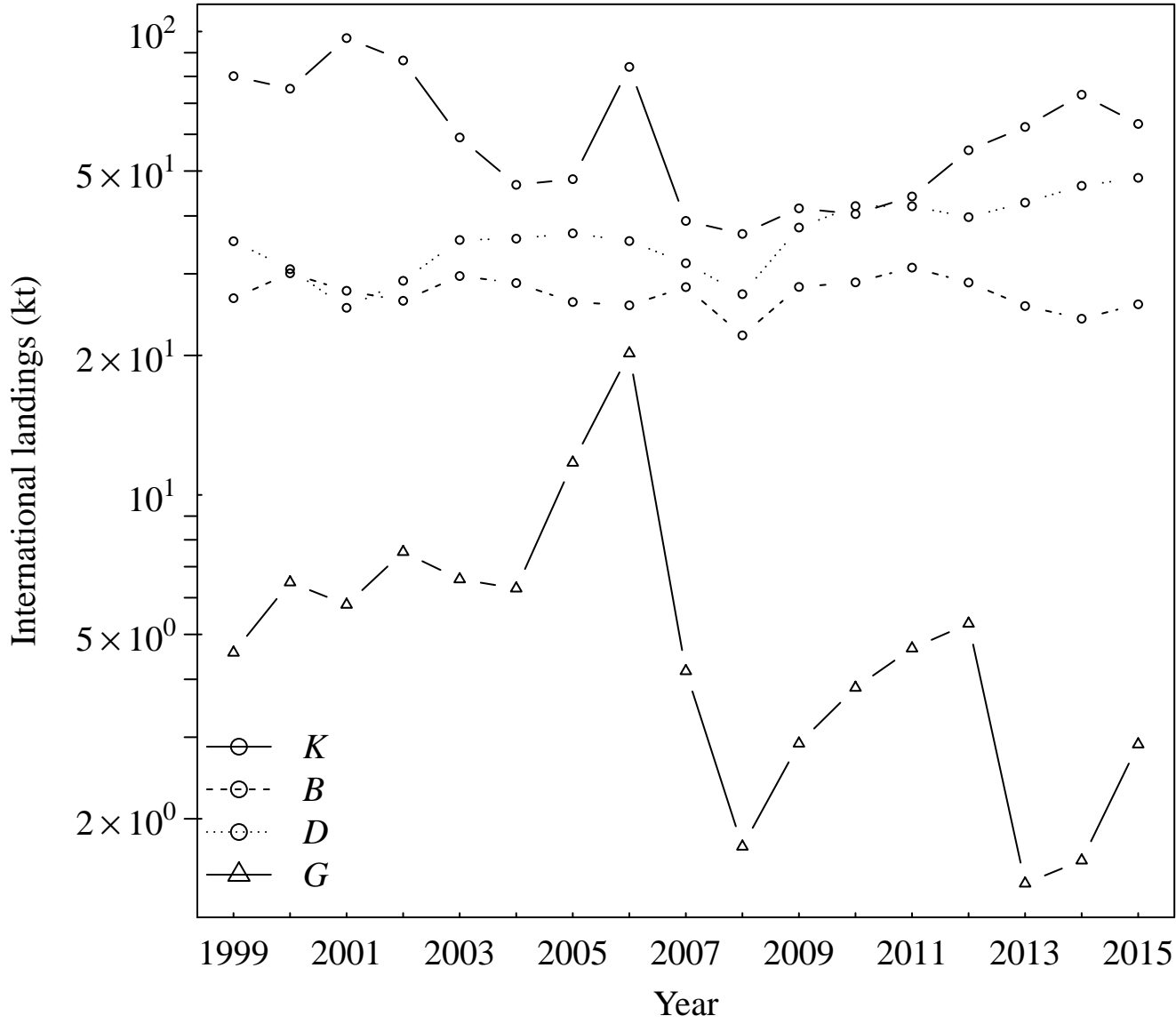

Supplement: Supplemental Information 9 [file peerj-07-7422-s009.pdf]

Stationary landings (kt)

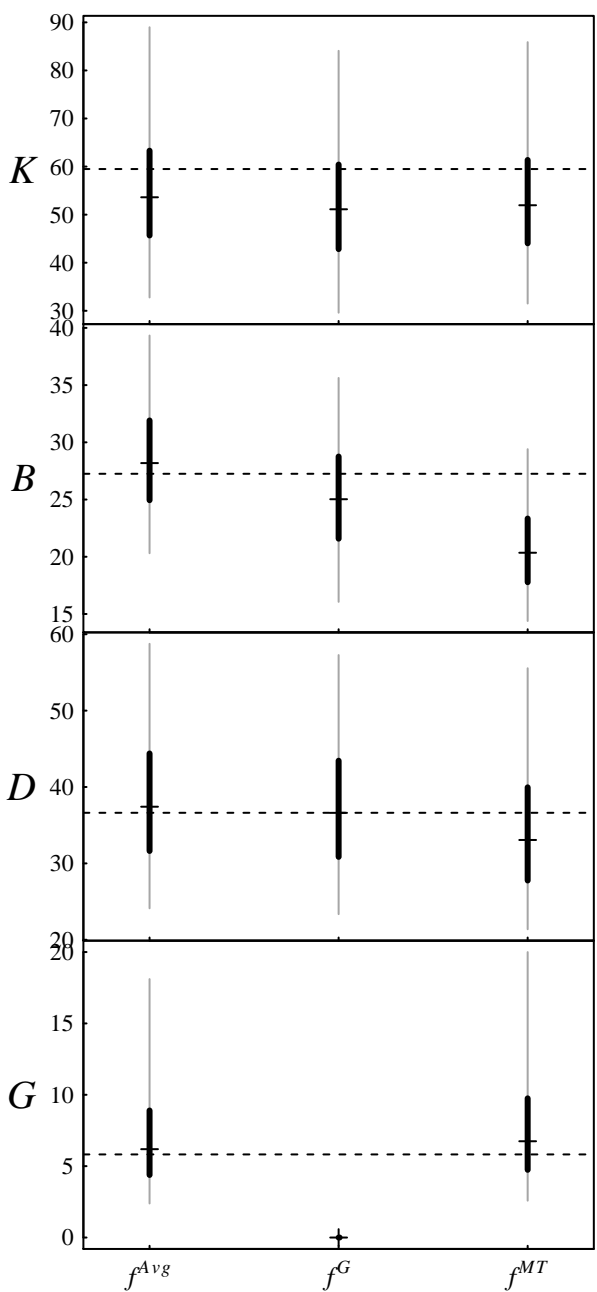

Supplement: Supplemental Information 10 [file peerj-07-7422-s010.pdf]

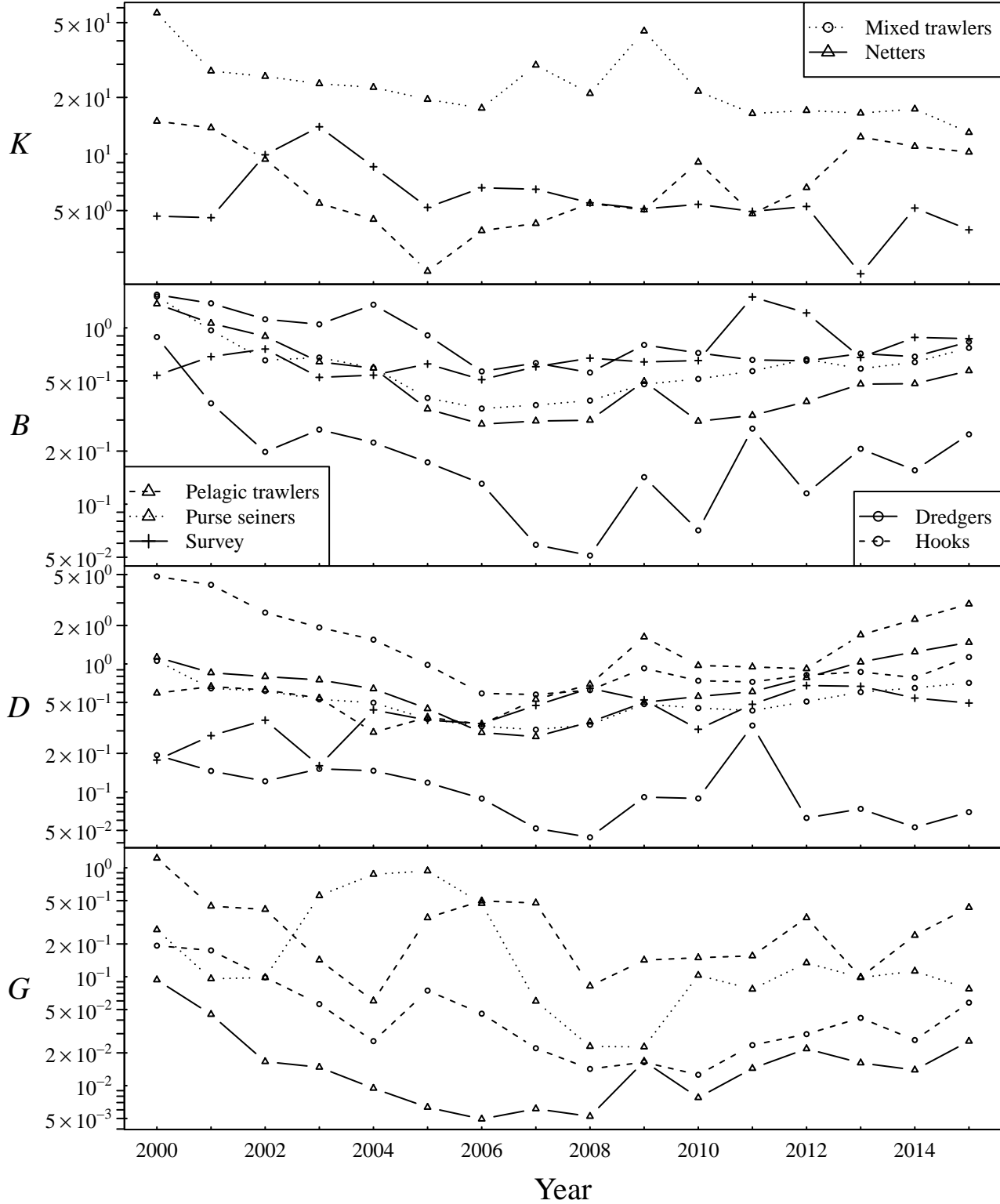

Supplement: Supplemental Information 11 [file peerj-07-7422-s011.pdf]
